# The mitochondrial permeability transition pore is a dispensable element for mitochondrial calcium efflux

**DOI:** 10.1016/j.ceca.2014.03.004

**Published:** 2014-07

**Authors:** Elena De Marchi, Massimo Bonora, Carlotta Giorgi, Paolo Pinton

**Affiliations:** Department of Morphology, Surgery and Experimental Medicine, Section of Pathology, Oncology and Experimental Biology, Interdisciplinary Center for the Study of Inflammation (ICSI), Laboratory for Technologies of Advanced Therapies (LTTA), University of Ferrara, Ferrara, Italy

**Keywords:** ANT, adenine nucleotide translocase, [Ca^2+^]c, cytosolic calcium concentration, [Ca^2+^]_m_, mitochondrial calcium concentration, CsA, cyclosporine A, HK, hexokinase II, H_2_O_2_, hydrogen peroxide, IB, intracellular milieu, MCU, mitochondrial Ca^2+^ uniporter, NCLX, mitochondrial Na^+^/Ca^2+^ antiporter, MPT, mitochondrial permeability transition, mPTP, mitochondrial permeability transition pore, PiC, inorganic phosphate carrier, PPIF, peptidyl prolyl isomerase, siRNAs, small-interfering RNAs, TSPO, peripheral benzodiazepine receptor, RuR, ruthenium red, VDAC, voltage-dependent anion channel, ATP5G1, Calcium (Ca^2+^), Cyclophilin F, Cyclosporine A (CsA), Mitochondria, Permeability transition pore (PTP), Peptidyl prolyl isomerase F (PPIF)

## Abstract

The mitochondrial permeability transition pore (mPTP) has long been known to have a role in mitochondrial calcium (Ca^2+^) homeostasis under pathological conditions as a mediator of the mitochondrial permeability transition and the activation of the consequent cell death mechanism. However, its role in the context of mitochondrial Ca^2+^ homeostasis is not yet clear. Several studies that were based on PPIF inhibition or knock out suggested that mPTP is involved in the Ca^2+^ efflux mechanism, while other observations have revealed the opposite result.

The c subunit of the mitochondrial F_1_/F_O_ ATP synthase has been recently found to be a fundamental component of the mPTP. In this work, we focused on the contribution of the mPTP in the Ca^2+^ efflux mechanism by modulating the expression of the c subunit. We observed that forcing mPTP opening or closing did not impair mitochondrial Ca^2+^ efflux. Therefore, our results strongly suggest that the mPTP does not participate in mitochondrial Ca^2+^ homeostasis in a physiological context in HeLa cells.

## Graphical abstract

**Figure:**
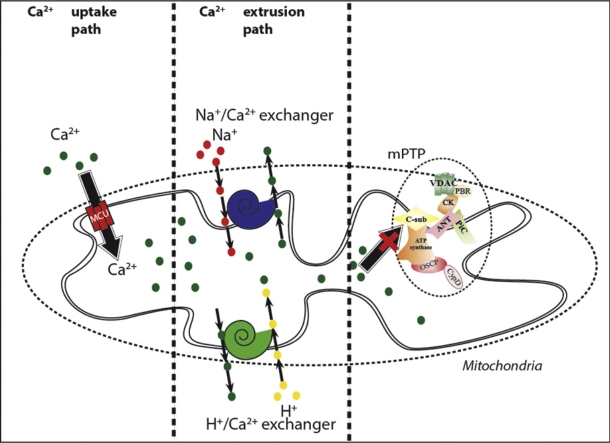

## 1 Introduction

Mitochondria are intracellular organelles involved in several cellular functions such as ATP production, fatty acid oxidation, Ca^2+^ signaling and cell death. [1–4]. Under pathological conditions, mitochondria are able to adapt by fusion or fission processes and modulate respiratory substrate utilization to regulate ATP production [5]. Furthermore, in cases of severe damage, mitochondria are eliminated by autophagy, and cell death can occur [6]. Thus, these organelles are master regulators of danger signaling [7,8].

A key signaling messenger that is able to transduce life or death signals to mitochondria is intracellular Ca^2+^ [9,10]. The mitochondrial Ca^2+^ uptake and release mechanisms are based on the utilization of gated channels for Ca^2+^ uptake and exchangers for release that are dependent upon the negative mitochondrial membrane potential, which represents the driving force for Ca^2+^ accumulation in the mitochondrial matrix [11]. The mitochondrial Ca^2+^ uniporter (MCU), which is encoded by the recently discovered gene ccdc109a [12,13], is responsible for Ca^2+^ influx, while the mitochondrial Na^+^/Ca^2+^ antiporter (NCLX) [14] is responsible for Ca^2+^ efflux. In spite of this information, the mitochondrial Ca^2+^ efflux mechanism has not been completely elucidated. It is mostly accepted that Na^+^/Ca^2+^ exchange activity is relevant for excitable cells [15] and that NCLX inhibition or silencing does not completely arrest Ca^2+^ efflux [14], which indicates that other mechanisms are involved in this process. Two mechanisms have been proposed to supplement Ca^2+^ efflux, one based on the H^+^/Ca^2+^ antiporter [16] and another based on the mitochondrial permeability transition pore (mPTP) [17], but evidence supporting the latter remains elusive.

The mPTP is a high-conductance channel that is located at the contact sites between the inner and outer mitochondrial membranes. This channel is responsible for the non-selective permeability state of the mitochondrial inner membrane. The transition to this state for small molecules is referred to as the mitochondrial permeability transition (MPT). The molecular composition of the mPTP is not yet clear, but several proteins have been shown to be components that participate in mPTP activity, including voltage-dependent anion channels (VDAC) [18], adenine nucleotide translocase (ANT) [19], the inorganic phosphate carrier (PiC), [20], peptidyl prolyl isomerase F (PPIF) [21,22], the peripheral benzodiazepine receptor (TSPO) [23], hexokinase II (HK) [24] and several members of the Bcl-2 family [25–27].

Ca^2+^ ions, prooxidant and proapoptotic proteins, a decrease in the mitochondrial membrane potential, pH variations and adenine nucleotides all sensitize the opening of the pore [28,29].

MPT resulting from mPTP opening is usually considered a transducer event in between Ca^2+^ or oxidative signal and different type of cell death [3,30]. Nonetheless several observations have suggested that mPTP is a component of the Ca^2+^ efflux mechanism [31–34] proposing a physiological role for this ambiguous complex. Unfortunately a different amount of studies have proposed the exact opposite [35,36] leaving this supposition still unresolved. Therefore, we focused on the role of the mPTP in mitochondrial Ca^2+^ homeostasis under non-pathological conditions. Recently, we suggested that, similar to PPIF, the c subunit of the F_O_ ATP synthase constitutes a critical component of the mPTP and that it is required for the MPT, mitochondrial fragmentation and cell death induced by oxidative stress or mitochondrial Ca^2+^ overload [37]. This concept was further confirmed by two different groups after our publication [38,39] and recently reviewed in [40].

By modulating c subunit expression and using pharmacological approaches, we revealed the important finding that the mPTP is not necessary for mitochondrial Ca^2+^ release; therefore, it does not participate in mitochondrial Ca^2+^ homeostasis in a non-pathological context, at least in HeLa cells.

## 2 Results

### 2.1 Regulation of mPTP activity by modulating the expression of the F_O_ ATP synthase c subunit

**Fig. 1:**
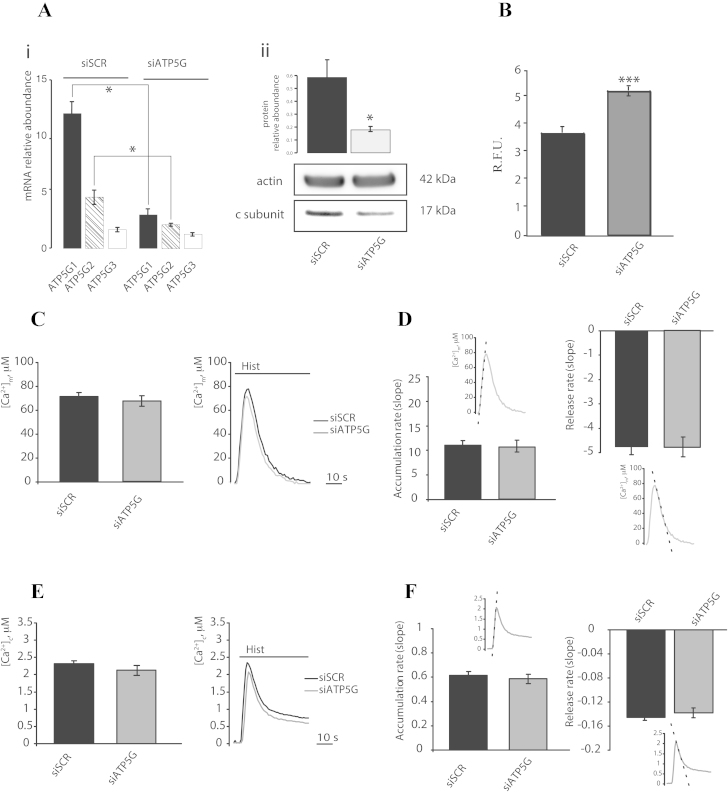
Calcium signaling during F_1_/F_O_ ATP synthase c subunit silencing in HeLa cells. The mRNA relative aboundance of ATP5G1, ATP5G2 and ATP5G3 (Ai) and the protein relative aboundance of ATP5G (Aii) after human cervical carcinoma (HeLa) cells transfection with a scrambled siRNA (siSCR) or a mix of siRNAs targeting ATP5G1, ATP5G2 and ATP5G3 (siATP5G) for 48 h (*n* = 3, independent experiments). Fluorescence intensity levels in the Calcein-Co^2+^ assay (B). HeLa cells were transfected as in (A) but in a combination with a plasmid encoding a mitochondrial red fluorescent protein (mtDsRED). These cells were monitored using fluorescence microscopy to assess the Calcein signal (*n* = 250, cells from 3 independent experiments). HeLa cells were transfected as in (A) but in a combination with a plasmid coding for a mitochondrial (C) (*n* = 30, from 6 indipendent experiments) or cytosolic (E) (*n* = 10, from 3 indipendent experiments) aequorin and then stimulated with 100 μM histamine (Hist). The rates of mitochondrial (D) and cytosolic (F) Ca^2+^ accumulation and release and a schematic representation of the meaning of the indexes are shown. The data are presented as means ± SEM; *p < 0.05,**p < 0.01.

To study the contribution of mPTP in mitochondrial Ca^2+^ homeostasis, we have investigated the kinetics of the mitochondrial and cytosolic Ca^2+^ influx and efflux. We have recently suggested that the c subunit of the F_1_/F_O_ ATP synthase is a component of the mPTP that is required for the MPT-driven mitochondrial fragmentation that is induced by cytosolic Ca^2+^ overload and oxidative stress [37]. Therefore, we decided to modulate mPTP activity by genetically manipulating the expression of the c subunit in the HeLa human cervical carcinoma cell line. The mammalian ATP synthase c subunit is encoded by three different nuclear genes (ATP5G1, ATP5G2 and ATP5G3) that produce three different protein products. These products differ in their mitochondrial localization sequence but result in the same mature protein after cleavage of the localization peptide [41]. As previously shown, we used a mix of three commercially validated small-interfering RNAs (siRNAs) to silence the three different genes that code for the c subunit (ATP5G1, ATP5G2 and ATP5G3). Analysis of the mRNA levels of the three genes using real-time PCR indicated that the silencing strategy resulted in an approximate reduction of 75% for ATP5G1, 53% for ATP5G2, and 20% for ATP5G3 (Fig. 1Ai), and western blot analysis showed that the protein levels were reduced to nearly 70% of the total protein level (Fig. 1Aii). To monitor the effective state of the mPTP, we used the calcein-Co^2+^ assay during c subunit silencing (Fig. 1B). As previously reported, ATP5G silencing ablated mPTP activity and resulted in increased fluorescence of mitochondrial calcein (Fig. 1B).

By taking advantage of aequorin technology [42], we first measured the mitochondrial and cytosolic Ca^2+^ concentrations after stimulation with 100 M histamine in HeLa cells that were transfected with a control siRNA (siSCR) or a mix of siRNAs that targeted ATP5G1, ATP5G2 and ATP5G3 (siATP5G) for 48 h (Fig. 1C). Silencing of the c subunit caused a non-significant reduction in Ca^2+^ uptake by the mitochondria (peak amplitude: 67.35 ± 4.41 μM vs. 71.08 ± 3.33 μM [siSCR]; *p* > 0.05) and in the Ca^2+^ uptake rate, but no significant variations in the rate of Ca^2+^ release from the mitochondria were observed (Fig. 1D). Furthermore, no significant differences in the cytosolic Ca^2+^ levels (peak amplitude: 2.13 ± 0.09 μM vs. 2.32 ± 0.08 μM [siSCR]; *p* > 0.05) (Fig. 1E) or in the kinetics of Ca^2+^ influx and efflux (Fig. 1F) were detected.

To confirm the experimental method, mitochondrial Ca^2+^ dynamics were monitored in the presence of a well-established blocker of the mitochondrial Ca^2+^ efflux system, CGP37157, which is an inhibitor of the mitochondrial Na^+^/Ca^2+^ exchanger [43].

HeLa cells were exposed to CGP37157 (10 μM) for 2 min, and Ca^2+^ uptake was induced with histamine (100 μM) in the continuous presence of the inhibitor.

CGP37157 caused an expected increase in Ca^2+^ uptake by the mitochondria (peak amplitude: 109.2 ± 15.52 μM vs. 68.6 ± 6.81 μM [Mock]; *p* < 0.05) (Fig. S1A) and a significant decrease of 31% in the rate of Ca^2+^ release from the mitochondria due to inhibition of the mitochondrial Na^+^/Ca^2+^ exchanger (Fig. S1B). The absence of significant differences between the cytosolic Ca^2+^ levels (peak amplitude: 2.84 ± 0.06 μM vs. 2.77 ± 0.33 μM [siSCR]; *p* > 0.05) (Fig. S1C) and Ca^2+^ influx and efflux kinetics (Fig. S1D) confirmed the specificity of CGP37157.

In our experimental settings, HeLa cells appeared to display higher RNA levels of ATP5G1 compared to its homologs. We thus decided to overexpress a Myc-tagged ATP5G1, which was under the control of the cytomegalovirus immediate early promoter, to verify its effect on mitochondrial Ca^2+^ homeostasis.

**Fig. 2:**
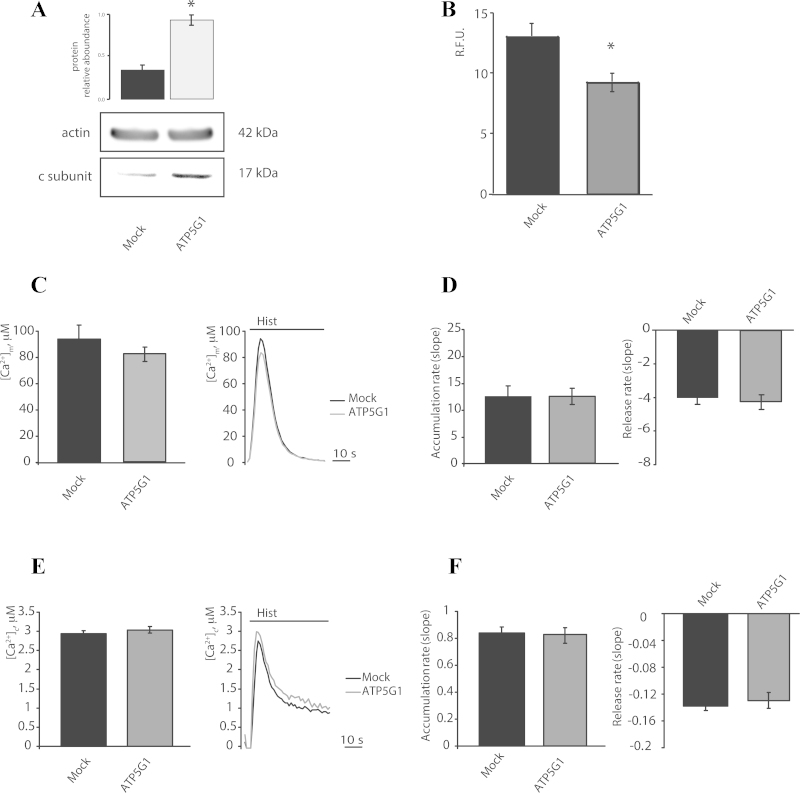
Calcium signaling during F_1_/F_O_ ATP synthase c subunit overexpression in HeLa cells. (A) The protein relative abundance of HeLa cells mock transfected or transfected for 48 h with a plasmid encoding MYC-tagged ATP5G1 (*n* = 3, independent experiments). Fluorescence intensity levels in the Calcein-Co^2+^ assay (B) in Hela cells transfected as in (A) but in a combination with a plasmid encoding mtDsRED (*n* = 15, cells from 3 independent experiments). Mitochondrial (C) and cytoplasmic (E) Ca^2+^ peak in HeLa cells transfected as in (B), in a combination with a plasmid coding for a mitochondrial (C) (*n* = 16 from 4 indipendent experiments) or cytosolic (E) (*n* = 10, from 3 indipendent experiments) aequorin, and then, the cells were stimulated with 100 μM histamine (Hist). Rates of mitochondrial (D) and cytosolic (F) Ca^2+^ accumulation and release. The data are presented as means ± SEM; **p < 0.01.

Overexperession levels were assessed by Western blot and results in dramatic increase in levels of c subunit (Fig. 2A). As expected, ATP5G1 overexpression was sufficient to induce the opening of the mPTP, as confirmed by the calcein-Co^2+^ assay (Fig. 2B).

Overexpression of the c subunit induces a tendency in lower the mitochondrial Ca^2+^ uptake (peak amplitude: 82.34 ± 5.4 μM vs. 93.89 ± 10.6 μM [Mock]; *p* > 0.05), without significantly affect the kinetics of Ca^2+^ influx and efflux (Fig. 2C and D) or cytosolic Ca^2+^ levels (peak amplitude: 3.03 ± 0.08 μM vs. 2.94 ± 0.07 μM [siSCR]; *p* > 0.05) (Fig. 2E and F).

### 2.2 Modulation by pharmacological treatments of mPTP activity in the regulation of mitochondrial Ca^2+^ influx/efflux

To confirm the absence of an effect of mPTP modulation on Ca^2+^ efflux, we used a pharmacological approach; specifically, Cyclosporine A (CsA) and hydrogen peroxide (H_2_O_2_) were used as an inhibitor of the mPTP [44] and an inducer of mPTP opening, respectively [45].

**Fig. 3:**
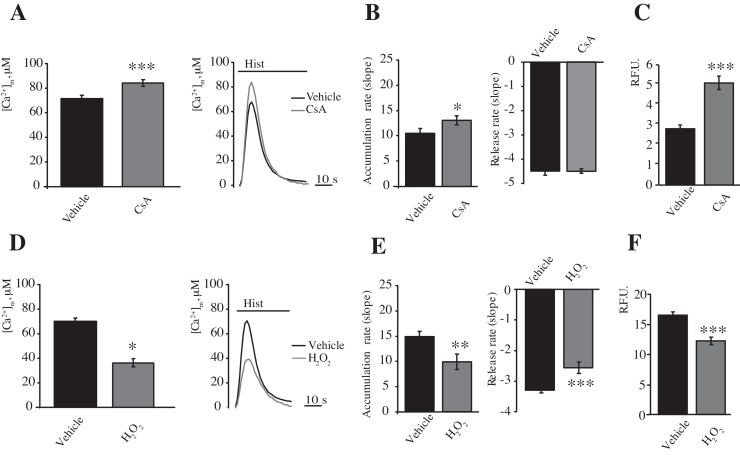
Modulation of the Ca^2+^ influx/efflux rates by CsA or H_2_O_2_ treatment. HeLa cells were transfected with a plasmid coding for a mitochondrial targeted aequorin and treated with 1 μM CsA for 30 min. The cells were then stimulated with 100 μM histamine (Hist) (A) (*n* = 16 from 4 independent experiments). Rates of mitochondrial (B) Ca^2+^ accumulation and release. (C) Fluorescence intensity levels in the Calcein-Co^2+^ assay of HeLa cells treated as in (A) but trasfected with a plasmid encoding a mitochondrial red fluorescent protein (mtDsRED). The cells were monitored by fluorescence microscopy to assess the Calcein signal (*n* = 25 cells from 3 independent experiments). HeLa cells were transfected with a plasmid coding for a mitochondrial aequorin and treated with 500 μM H_2_O_2_ for 30 min (D and E). The cells were then stimulated with 100 μM histamine (Hist) (D) (*n* = 12, from 3 indipendent experiments). Rates of mitochondrial (E) Ca^2+^ accumulation and release. Fluorescence intensity levels in the Calcein-Co^2+^ assay (F). HeLa cells were transfected with a plasmid encoding a mitochondrial red fluorescent protein (mtDsRED). The cells were monitored by fluorescence microscopy to assess the calcein signal (*n* = 40, from 3 independent experiments). The data are presented as means ± SEM; *p < 0.05, **p < 0.01, ***p < 0.001.

CsA (1 μM) was added to HeLa cells 30 min before stimulation with histamine (100 μM) and measurements of [Ca^2+^]_m_. As previously reported [37], CsA induced a significant increase in Ca^2+^ uptake (peak amplitude: 82.63 ± 2.82 μM vs. 70.2 ± 2.65 μM [vehicle]; *p* < 0.05) (Fig. 3A) and in the rate of Ca^2+^ accumulation in the mitochondria due to an increase in the mitochondrial membrane potential, which is the driving force for Ca^2+^ accumulation. In contrast, no differences in Ca^2+^ efflux kinetics (Fig. 3B) were observed. The closed state of the mPTP was confirmed using the calcein-Co^2+^ assay (Fig. 3C).

In contrast, to pharmacologically induce mPTP opening, HeLa cells were exposed to H_2_O_2_ (500 μM) 30 min before stimulation with histamine (100 μM). As expected, in the presence of H_2_O_2_, a significant decrease in Ca^2+^ uptake by the mitochondria was induced (peak amplitude: 36.52 ± 3.34 μM vs. 70.2 ± 2.65 μM [vehicle]; *p* < 0.001) (Fig. 3D).

Regarding the Ca^2+^ influx/efflux kinetics, a significant reduction of 33% in the rate of Ca^2+^ accumulation into the mitochondria and a significant reduction of 22% in the rate of Ca^2+^ release from the mitochondria in the presence of H_2_O_2_ (500 μM) occurred (Fig. 3E).

**Fig. 4:**
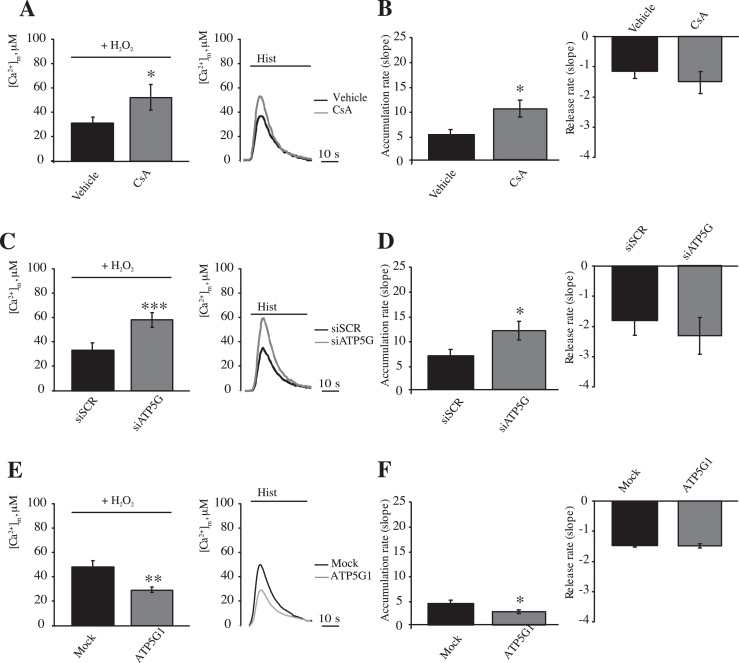
Modulation of the mPTP activity in combination with H_2_O_2_ treatment. HeLa cells were transfected with a plasmid coding for a mitochondrial targeted aequorin, treated with 1 μM CsA for 30 min and then treated with 500 μM H_2_O_2_ for 30 min (A and B). The cells were then stimulated with 100 μM histamine (Hist) (A) (*n* = 10, from 3 independent experiments). Rates of mitochondrial (B) Ca^2+^ accumulation and release. HeLa cells were transfected with a scrambled siRNA (siSCR) or a mix of siRNAs targeting ATP5G1, ATP5G2 and ATP5G3 (siATP5G) in a combination with a plasmid coding for a mitochondrial aequorin for 48 h and treated with 500 μM H_2_O_2_ for 30 min (C and D). The cells were then stimulated with 100 μM histamine (Hist) (C). (*n* = 10, from 3 independent experiments). Rates of mitochondrial (D) Ca^2+^ accumulation and release. HeLa cells were mock transfected or transfected for 48 h with a plasmid encoding MYC-tagged ATP5G1 in a combination with a plasmid coding for a mitochondrial aequorin and treated with 500 μM H_2_O_2_ for 30 min (E and F). The cells were then stimulated with 100 μM histamine (Hist) (E) (*n* = 10, from 3 independent experiments). Rates of mitochondrial Ca^2+^ accumulation and release (F). The data are presented as means ± SEM; *p < 0.05, ***p < 0.001.

To confirm the specificity of the effect of H_2_O_2_ on mPTP opening and Ca^2+^ handling, we combined H_2_O_2_ (500 μM) and CsA (1 μM) before stimulation with histamine (100 μM) (Fig. 4A). In such conditions, histamine challenge resulted in a partial recovery of the [Ca^2+^]_m_ (peak amplitude: 32.59 ± 5.79 μM [vehicle] vs. 51.6 ± 10.3 μM [CsA]; *p* < 0.05) and uptake rate, while no significant reduction in the kinetics of Ca^2+^ efflux were observed (Fig. 4B). Silencing of ATP5G resulted in protection of mPTP opening during challenge with the prooxidant H_2_O_2_ to an extent comparable with treatment with CsA [37]. As expected, HeLa cells depleted of ATP5G displayed a [Ca^2+^]_m_ that was protected from H_2_O_2_ in amplitude (peak amplitude: 32.6 ± 5.79 μM [siSCR] vs. 57.3 ± 4.96 μM [siATP5G]; *p* < 0.01) and uptake speed, while no significant differences were observed in the recovery phase of the peaks (Fig. 4C and D). In contrast, ATP5G1 overexpression resulted in the promotion of H_2_O_2_-induced suppression of mitochondrial Ca^2+^ uptake (peak amplitude: 47.9 ± 5.19 μM [Mock] vs. 29.2 ± 2.22 μM [ATP5G1]; *p* < 0.01), with a parallel inhibition of uptake and release kinetics (Fig. 4E and F).

Overall, the data show that the mPTP pharmacological opening inversely correlate with the amplitude and influx rate of [Ca^2+^]_m_, but does not affect efflux rates in living cells.

### 2.3 Mitochondrial Ca^2+^ homeostasis and mPTP modulation during saturation of calcium efflux system

Multiple authors have proposed that the mPTP exerts a significant effect on mitochondrial Ca^2+^ efflux only during saturation of the Ca^2+^ export system [46]. To explore this, we monitored the effect of genetic manipulation of ATP5G by blocking the mitochondrial Na^+^/Ca^2+^ exchanger.

**Fig. 5:**
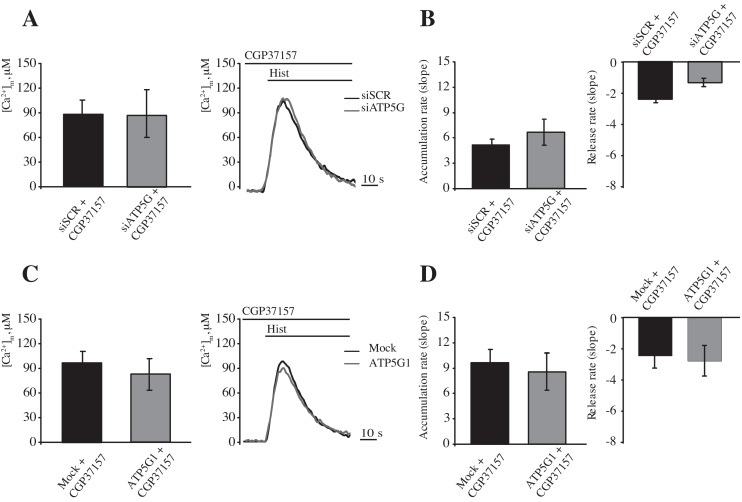
Modulation of the mPTP activity by combined CGP37157 exposure and c subunit silencing or c subunit overexpression. HeLa cells were transfected with a scrambled siRNA (siSCR) or a mix of siRNAs targeting ATP5G1, ATP5G2 and ATP5G3 (siATP5G) in a combination with a plasmid coding for a mitochondrial aequorin for 48 h and treated with 10 μM CGP37157 added 2 min before stimulation with histamine, which was done in the continuous presence of CGP37157 (A and B). The cells were then stimulated with 100 μM histamine (Hist) (A) (*n* = 10, from 3 independent experiments). Rates of mitochondrial Ca^2+^ accumulation and release (B). (C and D) HeLa cells were mock transfected or transfected for 48 h with a plasmid encoding MYC-tagged ATP5G1 in a combination with a plasmid coding for a mitochondrial aequorin and treated with 10 μM CGP37157 added 2 min before stimulation with histamine, which was done in the continuous presence of CGP37157. The cells were then stimulated with 100 μM histamine (Hist) (C) (*n* = 10, from 3 independent experiments). Rates of mitochondrial (D) Ca^2+^ accumulation and release (D). The data are presented as means ± SEM.

As previously shown (Fig. S1), exposure to CGP37157 induced a significant increase in the [Ca^2+^]_m_ during agonist stimulation with a concomitant reduction of the efflux rate. If mPTP opening extrudes Ca^2+^ only during the saturation of other efflux systems, then c subunit depletion should result in a strengthened inhibition of the efflux rate. However, silencing of ATP5G in HeLa cells exposed to CGP37157 did not display any significant differences in peak amplitude (Fig. 5A, peak amplitude: 86.2 ± 17.2 μM [siSCR] vs. 85.7 ± 15.93 μM [siATP5G]; *p* > 0.05), uptake, or release speed (Fig. 5B) when compared with siSCR-treated cells.

An analogous situation was observed during ATP5G1 overexpression. We proposed that ATP5G1 overexpression would sensitize MPT induction; therefore, the threshold for mPTP opening should be reached faster when efflux systems are saturated. Nonetheless, exposure to CGP37157 did not induce any significant variations in any of the measured indicators of mitochondrial Ca^2+^ homeostasis (Fig. 5C and D, peak amplitude: 95.23 ± 12.44 μM [Mock] vs. 81.27 ± 18.91 μM [ATP5G1]; *p* > 0.05).

**Fig. 6:**
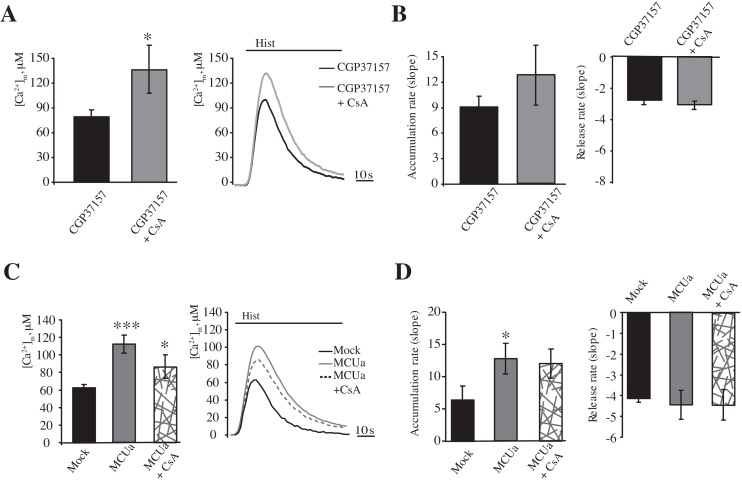
Modulation of the mPTP activity by CSA treatment in combination with CGP37157 treatment or MCUa overexpression. HeLa cells were transfected for 48 h with a plasmid coding for a mitochondrial aequorin and treated with 10 μM CGP37157 for 2 min, in absence or presence of 1 μM CsA for 30 min. (A and B). Ca^2+^ uptake was elicited by 100 μM histamine (Hist). (B) Rates of mitochondrial Ca^2+^ accumulation and release (*n* = 11, from 3 independent experiments). HeLa cells were mock transfected or transfected for 48 h with a plasmid encoding MCU-FLAG in a combination with a plasmid coding for a mitochondria-targeted aequorin in absence or presence of 1 μM CsA for 30 min (C and D). The cells were then stimulated with 100 μM histamine (Hist) (C) (*n* = 9, from 3 independent experiments). Rates of mitochondrial Ca^2+^ accumulation and release (D). The data are presented as means ± SEM; *p < 0.05, ***p < 0.001.

To avoid any aspecific effect related to modulation of ATP5G expression, the effect of efflux system saturation was tested using pharmacological inhibition of the mPTP by CsA. HeLa cells exposed to GCP37157 displayed a peak amplitude that was much lower than that in double-treated cells (Fig. 6A, peak amplitude: 132.2 ± 29.53 μM [CsA + CGP37157] vs. 75.84 ± 8.00 μM [CGP37157]; *p* < 0.05). This finding was also reflected in a dramatic increase in the uptake rate (Fig. 6B). Despite the different amounts of available Ca^2+^, CsA did not induce the hypothesized reduction in the efflux rate, but resulted in only a small increase (Fig. 6B).

Furthermore, as an alternative route to [Ca^2+^]_m_ saturation, we forced an increase in the [Ca^2+^]_m_ by overexpressing the MCU. HeLa cells overexpressing the mitochondrial Ca^2+^ uniporter displayed a dramatic increase in mitochondrial Ca^2+^ uptake (peak amplitude: 62.74 ± 3.69 μM vs. 112.14 ± 10.27 μM [Mock]; *p* < 0.01), which was near 100% (Fig. 6A). As expected, a significant stimulation of Ca^2+^ influx was observed (approximately 97%); however, no significant variations in efflux were detected (Fig. 6B). MCUa overexpression was then combined with mPTP inhibition using CsA. Cells that were exposed to CsA (1 μM) and overexpressing MCUa tended to display a reduction in the maximal [Ca^2+^]_m_, (peak amplitude: 112.2 ± 10.27 μM [MCUa] vs. 98.9 ± 13.57 μM [MCUa + CsA]; *p* > 0.05), which was in contrast to what was observed in mock-transfected cells (Figs. 3A and 6C). Nonetheless, no differences were detected in the Ca^2+^ efflux rates (Fig. 6D).

### 2.4 Effect of mPTP in mitochondrial Ca^2+^ homeostasis in permeabilized cells

**Fig. 7:**
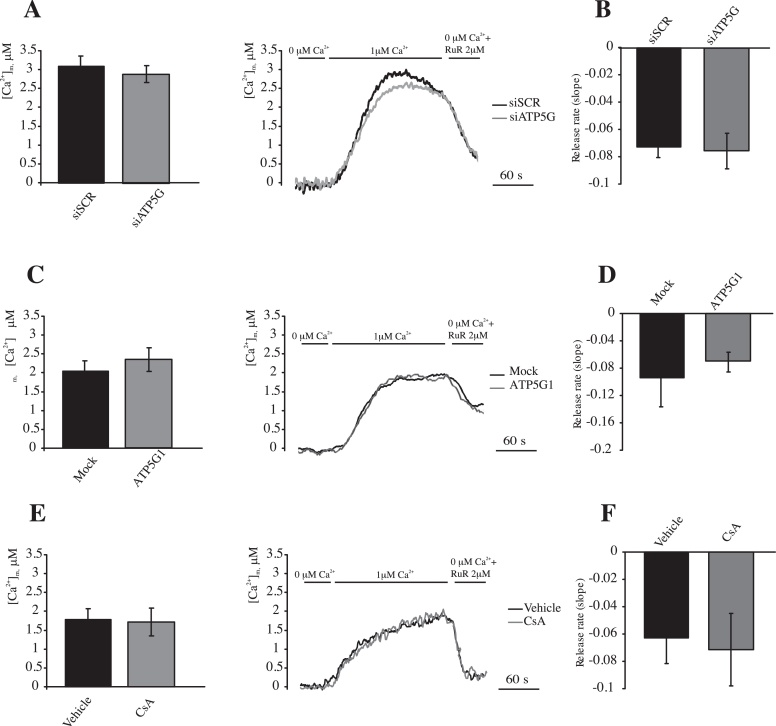
Modulation of the mPTP activity by c subunit silencing, c subunit overexpression and CsA treatment in permeabilized cells. (A and B) HeLa cells were transfected with a scrambled siRNA (siSCR) or a mix of siRNAs targeting ATP5G1, ATP5G2 and ATP5G3 (siATP5G) in a combination with a plasmid coding for a mitochondrial aequorin for 48 h (*n* = 9, from 3 indipendent experiments). (C and D) HeLa cells were mock transfected or transfected for 48 h with a plasmid encoding MYC-tagged ATP5G1 in a combination with a plasmid coding for a mitochondrial aequorin (*n* = 10, from 3 indipendent experiments). (E and F) HeLa cells were transfected with a plasmid coding for a mitochondria-targeted aequorin and treated with 1 μM CsA for 30 min or vehicle (*n* = 10 from 3 indipendent experiments). The cells were then digitonin-permeabilized and stimulated with 1 μM [Ca^2+^] in EGTA-buffered buffer. Calcium efflux was stimulated by perfusion with a calcium-free buffer supplemented with Ruthenium Red (RuR). Rate of mitochondrial Ca^2+^ release during Ca^2+^ deprivation and RuR perfusion (B, D and F). The data are presented as means ± SEM.

The efficiency of the mitochondrial Ca^2+^ efflux system has been proposed to be dependent upon [Ca^2+^]_m_. Some previously described conditions cause variations in the maximal [Ca^2+^]_m_. To avoid data misinterpretation due to this effect, we measured mitochondrial Ca^2+^ accumulation and release in permeabilized cells that were exposed to the same [Ca^2+^]. For this purpose, HeLa cells were perfused with a solution mimicking the intracellular milieu (IB) supplemented with 2 mM EGTA, the cells were then permeabilized with digitonin 30 μM for 1 min. The perfusion buffer was then changed to IB, which had an EGTA-buffered [Ca^2+^] of 1 μM. The cells were finally perfused with IB that was supplemented with Ruthenium Red (RuR), which is an inhibitor of the mitochondrial Ca^2+^ uniporter, and deprived of free Ca^2+^ to induce a maximal rate of Ca^2+^ release from the mitochondria. The same experiment was performed during modulation of the expression of the c subunit or in the presence of CsA (1 μM), which was added 30 min before the beginning of the measurements. The exposure of permeabilized cells transfected with scrambled siRNA to external [Ca^2+^] (1 μM) induced a rapid increase in the [Ca^2+^]_m_ that was stabilized at a value of 3.09 ± 0.24, which was analogous to the [Ca^2+^]_m_ that was reached in cells transfected with siATP5G (Fig. 7A, plateau 2.89 ± 0.22 μM). The switch to a Ca^2+^-free condition in the presence of RuR induced a dramatic decrease in the [Ca^2+^]_m_, and the rate at which this occurred appeared to be identical between cells transfected with scrambled siRNA or depleted for ATP5G (Fig. 7B). Negligible variations in the plateau levels or Ca^2+^ efflux rates were also detected when overexpressing ATP5G1 (Fig. 7C and D, plateau level: 2.03 ± 0.26 μM [Mock] vs. 2.34 ± 0.43 μM [ATP5G1]; *p* > 0.05) or during exposure to CsA (Fig. 7E and F, plateau level: 1.72 ± 0.28 μM [Vehicle] vs. 1.65 ± 0.74 μM [CsA]; *p* > 0.05). This finding confirmed that the mPTP does not affect the maximal mitochondrial Ca^2+^ efflux rate at the standard [Ca^2+^]_m_.

**Fig. 8:**
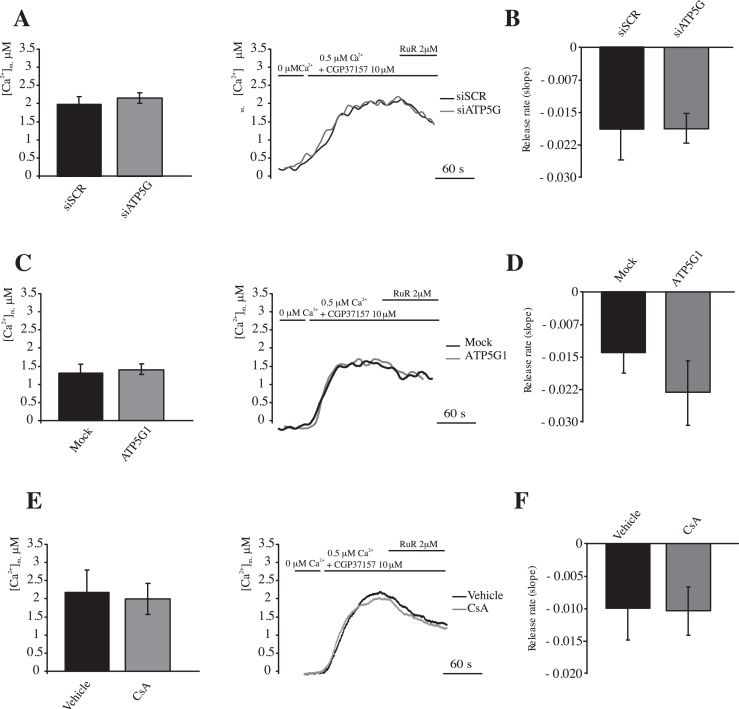
Modulation of the mPTP activity by combined CGP37157 and c subunit silencing, c subunit overexpression or CsA treatment in permeabilized cells. (A and B) HeLa cells were transfected with a scrambled siRNA (siSCR) or a mix of siRNAs targeting ATP5G1, ATP5G2 and ATP5G3 (siATP5G) in a combination with a plasmid coding for a mitochondrial aequorin for 48 h. (C and D) HeLa cells were mock transfected or transfected for 48 h with a plasmid encoding MYC-tagged ATP5G1 in a combination with a plasmid coding for a mitochondrial aequorin (*n* = 12, from 3 indipendent experiments). (E and F) HeLa cells were transfected with a plasmid coding for a mitochondria-targeted aequorin and treated with 1 μM CsA for 30 min or Vehicle (*n* = 9, from 3 indipendent experiments). The cells were then digitonin-permeabilized and stimulated with 0.5 μM [Ca^2+^] in EGTA-buffered buffer and in presence of CGP37157 10 μM. Calcium efflux was stimulated by 2 μM Ruthenium Red (RuR). Rate of mitochondrial Ca^2+^ release during RuR perfusion (B, D and F). The data are presented as means ± SEM.

Nonetheless, in such experimental conditions, a possible effect of the mPTP on efflux kinetics could be overcome by the activity of mNCX and the Ca^2+^/H^+^ antiporter. Therefore, the Ca^2+^ efflux rates were monitored in the presence of the mitochondrial Na^+^/Ca^2+^ exchanger CGP37157 and were elicited by 2 μM RuR and in the presence of external Ca^2+^. CGP37157 dramatically increased mitochondrial Ca^2+^ uptake, causing fast aequorin consumption when external Ca^2+^ 1 μM was added ([Ca^2+^]_m_ > 14 μM data not show). In this second group of permeabilized cells, Ca^2+^ uptake was elicited by adding external Ca^2+^ (0.5 μM). Administration of RuR induced slow (but not absent) Ca^2+^ release from the mitochondrial matrix in scrambled, siRNA-transfected HeLa cells (Fig. 8). Interestingly, cells transfected with siRNA for ATP5G1 displayed the same [Ca^2+^]_m_ at the plateau (Fig. 8A), and efflux speed in the presence of RuR (Fig. 8B). In parallel, the efflux rate in HeLa cells overexpressing ATP5G1 display a bare, but not significant, increase in Ca^2+^ efflux rate (Fig. 8C and D). Finally when the cells were pretreated with CsA (Fig. 8E and F), the extrusion rate appeared to be completely identical to that measured in untreated cells, indicating that mPTP was not responsible for the leakage of the remaining Ca^2+^ from the mitochondrial matrix.

## 3 Discussion

Correct Ca^2+^ uptake into the mitochondria is essential for mitochondrial oxidative phosphorylation, but excessive Ca^2+^ uptake leads to cell death [47]. Therefore, the balance between Ca^2+^ influx and efflux is fundamental for correct intracellular homeostasis and is extensively regulated by influx (through the mitochondrial Ca^2+^ uniporter) and efflux (through the Na^+^/Ca^2+^ or H^+^/Ca^2+^ exchanger) systems [35].

Some authors have proposed that the mPTP is a component of the Ca^2+^ efflux system due to the non-specific channel properties that were observed during its opening [17,34]. These reports are based mainly on pharmacological inhibition of the mPTP by cyclosporine A (CsA, which targets PPIF) or by knocking down PPIF, which causes typical alterations in mitochondrial Ca^2+^ homeostasis that could be associated with alterations in the mitochondrial Ca^2+^ efflux (especially alterations in the maximal [Ca^2+^]_m_ and Ca^2+^ extrusion rates). On the other hand, several other reports have indicated the opposite [35,36], which suggests a different function for CsA in affecting Ca^2+^ homeostasis.

Driven by our recent work suggesting that the c subunit of the F_1_/F_O_ ATP synthase is a component of the mPTP, we have focused our attention on the involvement of the mPTP in mitochondrial Ca^2+^ homeostasis under non-pathological conditions. The novelty of our study lies in the regulation of mPTP activity by manipulating the c subunit of the F_1_/F_O_ ATP synthase and measuring the [Ca^2+^]_m_ and [Ca^2+^]c using the pH-independent, luminescent, Ca^2+^-sensitive probe, aequorin [42].

Measurements of the [Ca^2+^]_m_ revealed a minimum reduction in mitochondrial Ca^2+^ loading after c subunit silencing, but no variations in the rate of Ca^2+^ release from the mitochondria were observed. Furthermore, no changes in the cytosolic Ca^2+^ levels were observed. As previously shown in this condition, we forced the closing of the mPTP, and the cells were protected from damage.

An analogous situation occurred when the c subunit was overexpressed, which forces the opening of the mPTP. In this case, mitochondrial Ca^2+^ accumulation was reduced, but no changes in the rate of Ca^2+^ influx or efflux occurred.

In both cases, the observed variations in peak amplitude correlated with a decrease in the mitochondrial membrane potential that is induced by c subunit silencing or overexpression, which we recently reported [37]. The overall modulation of c subunit expression suggested that, under physiological conditions in HeLa cells, mPTP is not directly involved in the Ca^2+^ efflux mechanism, and an indirect effect could be hypothesized instead.

We previously reported that, in this cell line, c subunit silencing generates a moderate reduction of the MMP, which is the opposite of the significant depolarization that occurs upon c subunit overexpression. Variations in the MMP, which is the driving force for Ca^2+^ accumulation in the mitochondrial matrix, could produce the exact effects on the [Ca^2+^]_m_ and uptake rate that were observed in this model. To validate these results, we took advantage of the well-known PPIF inhibitor CsA that was reported to be capable of increasing the mitochondrial membrane potential (MMP) and [Ca^2+^]_m_ [48,49]. After CsA treatment, a significant increase in mitochondrial Ca^2+^ uptake and the peak amplitude occurred, but, again, no differences in the rate of mitochondrial Ca^2+^ efflux were observed (which was in accordance with the observation after c subunit silencing).

However, we cannot completely ignore the several respectable reports proposing that there is an effect of the mPTP on mitochondrial Ca^2+^ efflux. Most of these reports were based on PPIF inhibition or knock out. It could then be speculated that PPIF could somehow affect other participants of the Ca^2+^ efflux system, as proposed by Wei and co-workers [35], and possibly generate a conformational rearrangement upon interaction with other proteins due to its peptidyl-prolyl isomerase activity.

It could be argued that c subunit overexpression did not lead to an mPTP opening event sufficient to generate a detectable Ca^2+^ efflux. To test this hypothesis, we induced mPTP opening through the use of the prototypic, pro-oxidant hydrogen peroxide. Exposure to this stress agent results in mPTP opening with a consequent reduction in the maximal [Ca^2+^]_m_ after agonist exposure and an increase in calcein quenching by cobalt. In this condition, a decrease in the mitochondrial Ca^2+^ influx and efflux were observed, which was in agreement with the reduced driving force, while an opened, non-specific Ca^2+^ channel (such as the mPTP) should produce a significant increase in the Ca^2+^ efflux rate.

Nonetheless, although the [Ca^2+^]_m_ was partially recovered in cells silenced for ATP5G or pretreated with CsA, protection from the mPTP did not modify the kinetics of Ca^2+^ efflux. In contrast, overexpression of ATP5G1 promoted H_2_O_2_-induced mPTP opening, causing a further reduction in the maximal [Ca^2+^]_m_ under agonist stimulation. In spite of this reduction, the efflux rate did not display a significant stimulation, but rather a small inhibition. These observations provide strong confirmation that, even following potent induction of its opening, the mPTP does not participate in Ca^2+^ efflux. We previously confirmed that induction of the mPTP by H_2_O_2_ caused slow MMP dissipation (probably due to the induction of the low conductance mode of mPTP) [37,50]. During protection or induction of mPTP opening, the [Ca^2+^]_m_ recovering phase seems to correlate with the levels of MMP previously observed in such conditions [37], thus strengthening the hypothesis that a possible modulation of the [Ca^2+^]_m_ by the mPTP should indirectly occur due to regulation of the MMP.

It was proposed that the effects of the mPTP on the kinetics of Ca^2+^ efflux could be only relevant as a “safe valve” in mitochondria with a saturated Ca^2+^ efflux system [46], which is analogous to what was observed in the Ca^2+^ retention capacity experiments that largely used isolated mitochondria to determine the threshold for mPTP opening. To challenge this hypothesis in living cells, we combined c subunit expression with blockade of the mitochondrial Na^+^/Ca^2+^ antiporter through the use of CGP37157. Interestingly, all the previously observed tendencies that were observed after silencing or overexpressing the c subunit were completely masked by the use of CGP37157, suggesting that sustained [Ca^2+^]_m_ levels are not sufficient to induce a calcium efflux through the mPTP. Furthermore, an effect of on the [Ca^2+^]_m_ was still observable as an increase in the [Ca^2+^]_m_ and influx rate when blocking mNCX (characters dependent on the MMP); nonetheless, the efflux rate was still not affected.

An alternative Ca^2+^ overload approach was proposed. Recently, several components of the mitochondrial Ca^2+^ uptake machinery have been revealed [51]. The core element of the MCU system is the product of the CCDC109a gene (MCUa [12,13]). Overexpressing the MCUa, as expected, resulted in an extraordinary increase in the Ca^2+^ influx rate and [Ca^2+^]_m_. Such an increase was not further stimulated by inhibition of the mPTP opening through treatment with CsA, which suggested that the mitochondrial network was near its maximal uptake capacity. Interestingly, MCUa overexpression also resulted in a reproducible tendency to increase the Ca^2+^ efflux rate. Treatment with CsA did not generate any significant effects on the calcium efflux rates of MCUa-overexpressing cells. Overall, these results clearly indicate that overload of the [Ca^2+^]_m_ in our cell model is not sufficient to induce a mPTP-dependent Ca^2+^ efflux.

The Ca^2+^ efflux system is dependent upon [Ca^2+^]_m_, which is exposed; and modulation of the expression of the c subunit as well as exposure to CsA results in alterations of the [Ca^2+^]_m_.

To avoid misinterpretation of the data due to different values for the [Ca^2+^]_m_, Ca^2+^ efflux measurements were performed in permeabilized cells that had been stimulated with external Ca^2+^ (1 μM) to obtain a stable level of [Ca^2+^]_m_ in the control mitochondria. After that plateau was reached, the external Ca^2+^ was removed, and the MCU inhibitor Ruthenium Red was added to avoid Ca^2+^ reuptake. The rate of Ca^2+^ release from the mitochondria under these conditions was considered the maximum efflux speed that could be reached, and no significant differences in the rate after mPTP inhibition with CsA or during ATP5G silencing or its overexpression were observed. It is reasonable to assume that because mitochondria are energized by the addition of ADP, pyruvate, and succinate in permeabilized cells, the efflux rate is driven mostly by the mNCX and the H^+^/Ca^2+^ exchangers.

Nonetheless, further induction of efflux system saturation by inhibiting the mitochondrial Na^+^/Ca^2+^ antiporter (in the presence of external Ca^2+^) and MCU (by RuR) generated a Ca^2+^ leak from the mitochondrial matrix. This leak was independent of mPTP inhibition (by ATP5G silencing or CsA administration) and stimulation (by ATP5G1 overexpression) and was thus ascribable only to the H^+^/Ca^2+^ exchanger. These results exclude the possible involvement of the mPTP as a component of the Ca^2+^ efflux system, even when standardized Ca^2+^ concentrations where achieved.

The presented results suggest that, in spite of what can be observed in isolated mitochondria, the mPTP has a Ca^2+^-induction threshold that is too high to be reached in living cells, and this is most likely due to the high efficiency of the Na^+^/Ca^2+^ and H^+^/Ca^2+^ exchangers, which can could compete with and overcome the mPTP. It should also be considered that the Ca^2+^ efflux from mitochondria is an energetically unfavorable process due to the strong electrochemical gradient, which is negative in the matrix. This allows for passive import into the matrix, as occurs during MCU. If the current model for the mPTP, which states that it forms a nonspecific channel, is correct, then its partial opening, even in the presence of reduced MMP (as it apparently occurs in its low-conductance mode), should favor import instead of efflux. In contrast, efflux through a channel would be expected on de-energized mitochondria, as was reported for the MCU [52]. Still has to be considered that inhibition of mPTP induce increase in Ca^2+^ loading capacity that can lead to generation of toxic Ca^2+^ phosphorous rich precipitates [53]. Present results do not exclude mPTP participation in this phenomenon, but it would suggest that it could be indirectly involved. Opening of mPTP do not directly allow Ca^2+^ transport, but rather would dissipated mitochondrial membrane potential facilitating Ca^2+^ efflux by inverse transportation through MCU.

Altogether, these data demonstrate that, under physiological conditions, the mPTP is a dispensable element for mitochondrial Ca^2+^ homeostasis and, especially, for mitochondrial Ca^2+^ release at least in our cell model.

## 4 Materials and methods

### 4.1 Chemicals, cell cultures and transfection

Human cervical carcinoma HeLa cells were maintained in a humidified 5% CO_2_, 37 °C incubator in Dulbecco's modified Eagle's medium (DMEM) supplemented with 10% fetal bovine serum (FBS), 100 U/ml penicillin (EuroClone, 3001D), 100 μg/ml streptomycin (EuroClone, 3000D).

RNA interference experiments were performed by transfecting cells with a commercial control siRNA (AllStars RNAi Controls, herein referred to as siSCR), with a mix of siRNAs targeting ATP5G1 (5′-GCU CUG AUC CGC UGU UGU AdTdT-3′), ATP5G1 (5′-CGG AGA UAC UGA CAG AUG AdTdT-3′) and ATP5G3 (5′-AGG GCU CUA CGG UAU UUA AdTdT-3′), all purchased from Qiagen. siRNAs were transfected by means of the HiPerfect^®^ transfection reagent, as per manufacturer's instructions [54].

For transient overexpression experiments, a pCMV6 entry- based plasmid coding for a MYC-tagged variant of ATP5G1 under the control of the CMV immediate early promoter (RC200292) was obtained from OriGene, A pcDNA3 based plasmid coding for MCU-flag tagged was gift from prof. Rosario Rizzuto from University of Padua, pcDNA3 empty vector was used as Mock. Plasmid transfections were performed via the standard Ca^2+^ phosphate procedure [55].

### 4.2 RNA extraction and gene expression analyses

For the expression analysis of *ATP5G1*, *ATP5G2*, and *ATP5G3* silencing, total RNA was purified from HeLa cells transfected with scrambled or ATP5G1 siRNAs (siATP5G1, 2 and 3), for 48 h following the standard TRIzol protocol. To analyze mRNA expression, qRT-PCR was performed on 500 ng of total RNA using oligo dT (Fermentas) and random primers (Gibco). Quantitative PCR reaction was performed using Qiagen Taq DNA Polymerase and EvaGreen (Biotium Inc.). The following oligonucleotides were used as primers for the qPCR reaction: *ATP5G1*-fw, 5′-GCTGAGACCAAGGGCTAAAG-3′; *ATP5G1*-rv, 5′-CGGATCAGAGCTGGAGAAATG-3′; *ATP5G2*-fw, 5′-TGGAACTGTGTTTGGGAGC-3′; *ATP5G2*-rv, 5′-TGGCAAAGAGGATGAGAAAGG-3′; *ATP5G3*-fw, 5′-TGGTTCTGGTGCTGGTATTG-3′; *ATP5G3*-rv, 5′-GAGACCCATAGCTTCAGACAAG-3′.

Reactions were incubated in a 96-well PCR plate at 95 °C for 15 min, followed by 40 cycles of 95° C for 30 s and 58 °C for 1 min, on Bio-Rad-Chromo4 Real Time thermal Cycler.

(available at http://fiji.sc/) and the parallel iterative deconvolution plugin.

## 5 Western blot

48 h after transfection cells were washed, harvested and lysed in RIPA buffer (50 mM Tris–HCl pH 7.8, 150 mM NaCl, 1% IGEPAL CA-630, 0.5% sodium deoxycholate, 0.1% SDS, 1 mM DTT) supplemented with proteases and phosphatases inhibitors (2 mM Na_3_VO_4_, 2 mM NaF, 1 mM PMSF and Protease Inhibitor Cocktail). Proteins were quantified using the Bradford Protein Assay (Bio-Rad), and separated on Novex NuPage Bis–Tris 4–12% precast gel (Invitrogen) and transferred to PVDF (BioRad) membranes for standard western blotting.

Immunoblotting of the membranes was performed using the following primary antibodies: anti-ATP synthase C antibody (1:300, Abcam) and anti-actin (1:5000, Sigma–Aldrich). Signals were revealed after incubation with recommended secondary antibody coupled to peroxidase by using enhanced chemiluminescence.

### 5.1 Aequorin measurements

Probes used are chimeric aequorins targeted to the cytosol (cytAEQ) and mitochondria (mtAEQmut). “AEQ” refers to wild-type aequorin, and “AEQmut” refers to a low affinity D119A mutant of aequorin. For the experiments with cytAEQ and mtAEQmut, cells were incubated with 5 mM coelenterazine (Fluka, 7372) for 1–2 h in Krebs–Ringer buffer (KRB). A coverslip with transfected cells was placed in a perfused thermostated chamber located in close proximity to a low-noise photomultiplier with a built-in amplifier/discriminator.

All aequorin measurements were performed in KRB supplemented with 1 mM CaCl_2_. Agonist was added to the same medium as specified in figure legends. All experiments were terminated by lysing cells with 100 μM Triton in a hypotonic Ca^2+^-containing solution (10 mM CaCl_2_ in H_2_O) thus discharging the remaining aequorin pool. The output of the discriminator was captured by a Thorn EMI photon-counting board and stored in an IBM-compatible computer for further analyses. The aequorin luminescence data were calibrated offline into [Ca^2+^] values using a computer algorithm based on the Ca^2+^ response curve of wild-type and mutant aequorins.

In the experiments with permeabilized cells, a buffer mimicking the cytosolic ionic composition, (intracellular buffer) was used: 130 mM KCl, 10 mM NaCl, 2 mM K_2_HPO_4_, 5 mM succinic acid, 5 mM malic acid, 1 mM MgCl_2_, 20 mM HEPES, 1 mM pyruvate, 0.5 mM ATP (pH 7 at 37 °C). Intracellular buffer was supplemented with 2 mM EGTA and 2 mM HEDTA-buffered [Ca^2+^] 1 μM or 0.5 μM (intracellular buffer/Ca^2+^), calculated with the Chelator software [56]. HeLa cells were permeabilized by a 1-min perfusion with 50 μM digitonin (added to intracellular buffer/EGTA) during luminescence measurements. Mitochondrial Ca^2+^ uptake rate was calculated as the first derivative by using Microsoft Excel (Microsoft Co.); all the slopes were calculated at the same [Ca^2+^]_m_ to ensure comparable activity of efflux system.

### 5.2 Calcein-Co^2+^ assays

At day one HeLa Cells were seeded on 25 mm glass coverslip. After 24 h were transfected with Hyperfect or standard Ca^2+^ phosphate procedure as previously described, a plasmid coding for mtDsRed was added to the transfection for all procedures. After 36 h of expression HeLa were loaded with 1 mM Calcein acetoxymethyl ester and Co^2+^ as instructed by the Image-IT^®^ LIVE Mitochondrial Transition Pore Assay Kit (Molecular Probes-Life Technologies). Cells were then imaged by means of 490 ± 20 nm excitation and 525 nm long pass emission filters on a Axiovert 200 M fluorescence microscope equipped with a 40× water immersion objective (N.A. 1.2, from Carl Zeiss). Finally, images were analyzed with a custom Cell Profiler pipeline (http://www.cellprofiler.org/), briefly, after a background normalization Calcein average intensity was calculated over the mtDsRed mask.

### 5.3 Statistical procedures

Data were expressed as the mean ± SEM and were analyzed with Microsoft Excel (Microsoft Co.). Statistical significance was determined by means of ANOVA followed by two-tailed unpaired Student's *t*-tests. *p* values < 0.05 were considered statistically significant.
