# Supplementary material for: The mitochondrial permeability transition pore is a dispensable element for mitochondrial calcium efflux
Source: Cell Calcium. 2014 Jul;56(1):1–13. doi: 10.1016/j.ceca.2014.03.004 (PMC4074345; doi:10.1016/j.ceca.2014.03.004)
Supplement: Supplementary file 1 [file mmc1.pdf]

SUPPLEMENTARY FIGURE1

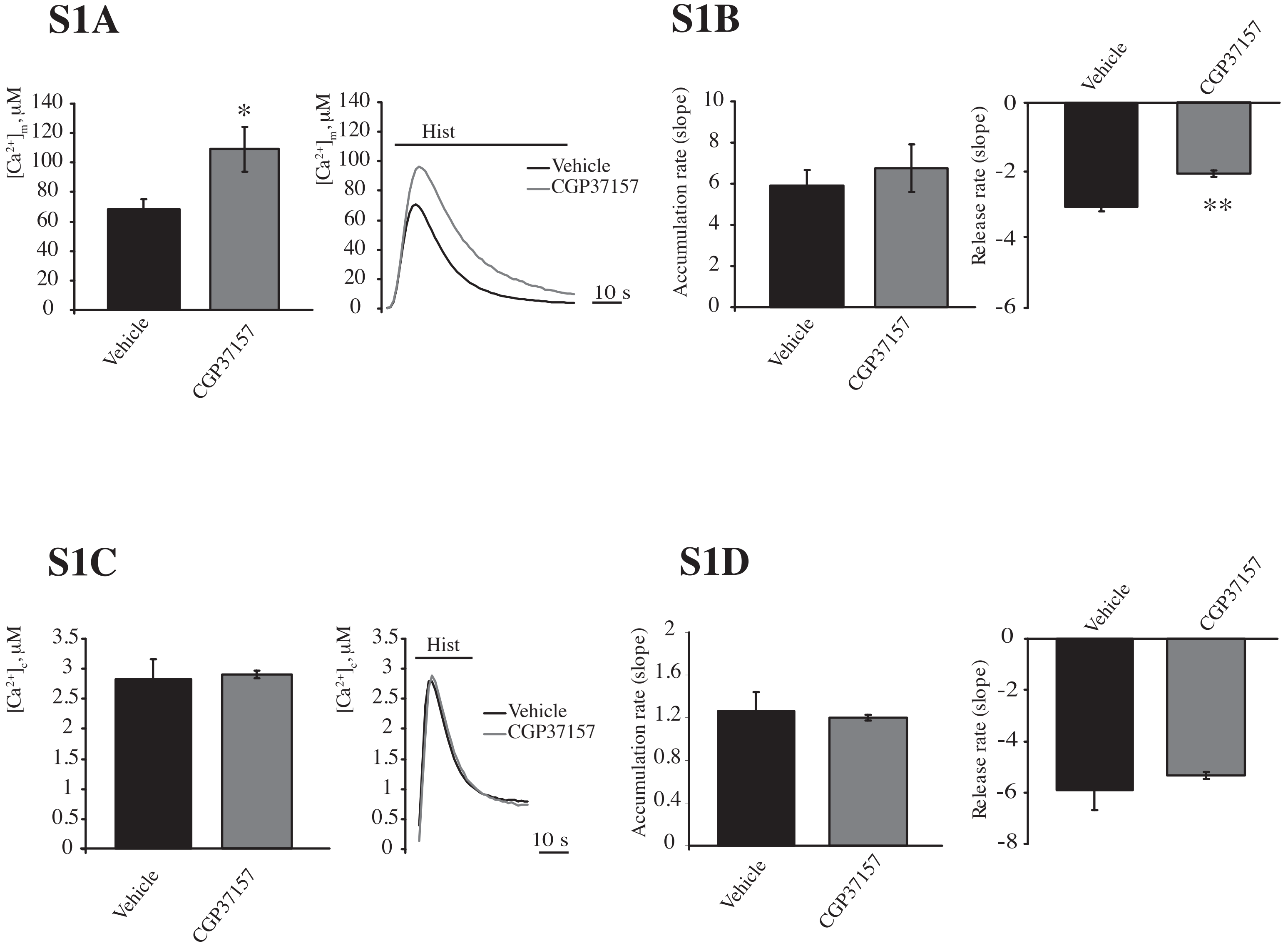

SUPPLEMENTARY FIGURE. Inhibition of mitochondrial Ca2+ efflux by CGP37157. HeLa cells were transfected with a plasmid coding for a mitochondrial (**S1A** and **B**; n=14, from 3 independent experiments) or cytosolic (**S1C** and **S1D**; n=10, from 3 independent experiments) aequorin and treated with 10 μM CGP37157 for 2 min. The cells were then stimulated with 100 μM histamine (Hist) in the continuous presence of the inhibitor.

Rates of mitochondrial (**S1B**) or cytosolic (**S1D**) Ca^{2+} accumulation and release. The data are presented as means ± SEM *$p < 0.05$.
